# Pragmatic approaches to analyzing qualitative data for implementation science: an introduction

**DOI:** 10.1186/s43058-021-00174-1

**Published:** 2021-06-29

**Authors:** Shoba Ramanadhan, Anna C. Revette, Rebekka M. Lee, Emma L. Aveling

**Affiliations:** 1 Department of Social and Behavioral Sciences, Harvard T.H. Chan School of Public Health, Boston, MA 02115 USA; 2 Division of Population Sciences, Dana-Farber Cancer Institute, 450 Brookline Ave, Boston, MA 02215 USA; 3 Department of Health Policy and Management, Harvard T.H. Chan School of Public Health, Boston, MA 02115 USA

**Keywords:** Implementation science, Qualitative, Methods, Pragmatic, Analysis, Practice-based

## Abstract

Qualitative methods are critical for implementation science as they generate opportunities to examine complexity and include a diversity of perspectives. However, it can be a challenge to identify the approach that will provide the best fit for achieving a given set of practice-driven research needs. After all, implementation scientists must find a balance between speed and rigor, reliance on existing frameworks and new discoveries, and inclusion of insider and outsider perspectives. This paper offers guidance on taking a pragmatic approach to analysis, which entails strategically combining and borrowing from established qualitative approaches to meet a study’s needs, typically with guidance from an existing framework and with explicit research and practice change goals.

Section 1 offers a series of practical questions to guide the development of a pragmatic analytic approach. These include examining the balance of inductive and deductive procedures, the extent to which insider or outsider perspectives are privileged, study requirements related to data and products that support scientific advancement and practice change, and strategic resource allocation. This is followed by an introduction to three approaches commonly considered for implementation science projects: grounded theory, framework analysis, and interpretive phenomenological analysis, highlighting core analytic procedures that may be borrowed for a pragmatic approach. Section 2 addresses opportunities to ensure and communicate rigor of pragmatic analytic approaches. Section 3 provides an illustrative example from the team’s work, highlighting how a pragmatic analytic approach was designed and executed and the diversity of research and practice products generated.

As qualitative inquiry gains prominence in implementation science, it is critical to take advantage of qualitative methods’ diversity and flexibility. This paper furthers the conversation regarding how to strategically mix and match components of established qualitative approaches to meet the analytic needs of implementation science projects, thereby supporting high-impact research and improved opportunities to create practice change.

Contributions to the literature

- Qualitative methods are increasingly being used in implementation science, yet many researchers are new to these methods or unaware of the flexibility afforded by applied qualitative research.
- Implementation scientists can benefit from guidance on creating a pragmatic approach to analysis, which includes the strategic combining and borrowing from established approaches to meet a given study’s needs, typically with guidance from an implementation science framework and explicit research and practice change goals.
- Through practical questions and examples, we provide guidance for using pragmatic analytic approaches to meet the needs and constraints of implementation science projects while maintaining and communicating the work’s rigor.

## Background

Implementation science (IS) is truly pragmatic at its core, answering questions about how existing evidence can be best translated into practice to accelerate impact on population health and health equity. Qualitative methods are critical to support this endeavor as they support the examination of the dynamic context and systems into which evidence-based interventions (EBIs) are integrated — addressing the “hows and whys” of implementation [1]. Numerous IS frameworks highlight the complexity of the systems in which implementation efforts occur and the uncertainty regarding how various determinants interact to produce multi-level outcomes [2]. With that lens, it is unsurprising that diverse qualitative methodologies are receiving increasing attention in IS as they allow for an in-depth understanding of complex processes and interactions [1, 3, 4]. Given the wide variety of possible analytic approaches and techniques, an important question is *which* analytic approach best fits a given set of practice-driven research needs. Thoughtful design is needed to align research questions and objectives, the nature of the subject matter, the overall approach, the methods (specific tools and techniques used to achieve research goals, including data collection procedures), and the analytic strategies (including procedures used for exploring and interpreting data) [5, 6]. Achieving this kind of alignment is often described as “fit,” “methodological integrity,” or “internal coherence” [3, 7, 8]. Tailoring research designs to the unique constellation of these considerations in a given study may also require creative adaptation or innovation of analytic procedures [7]. Yet, for IS researchers newer to qualitative approaches, a lack of understanding of the range of relevant options may limit their ability to effectively connect qualitative approaches and research goals.

For IS studies, several factors further complicate the selection of analytic approaches. First, there is a tension between the speed with which IS must move to be relevant and the need to conduct rigorous research. Second, though qualitative research is often associated with attempts to generate new theories, qualitative IS studies’ goals may also include elaborating conceptual definitions, creating classifications or typologies, and examining mechanisms and associations [9]. Given the wealth of existing IS frameworks and models, covering determinants, processes, and outcomes [10], IS studies often focus on extending or applying existing frameworks. Third, as an applied field, IS work usually entails integrating different kinds of “insider” and “outsider” expertise to support implementation or practice change [11]. Fourth, diverse traditions have contributed to the new field of IS, including agriculture, operations research, public health, medicine, anthropology, sociology, and more [12]. The diversity of disciplines among IS researchers can bring a wealth of complementary perspectives but may also pose challenges in communicating about research processes.

Pragmatic approaches to qualitative analysis are likely valuable for IS researchers yet have not received enough attention in the IS literature to support researchers in using them confidently. *By pragmatic approaches, we mean strategic combining and borrowing from established qualitative approaches to meet the needs of a given IS study, often with guidance from an IS framework and with clear research and practice change goals.* Pragmatic approaches are not new, but they receive less attention in qualitative research overall and are not always clearly explicated in the literature [9]. Part of the challenge in using pragmatic approaches is the lack of guidance on how to mix and match components of established approaches in a coherent, credible manner.

Our motivation in offering this guidance reflects our experiences as researchers, collaborators, and teachers connecting qualitative methods and IS research questions. The author team includes two behavioral scientists who conduct stakeholder-engaged implementation science and regularly utilize qualitative approaches (SR and RL). The team also includes a sociologist and a social psychologist who were trained in qualitative methods and have rich expertise with health services and implementation research (AR and EA). Through conducting qualitative IS studies and supporting students and colleagues new to qualitative approaches, we noticed a regularly occurring set of concerns and queries. Many questions seem to stem from a sense that there is a singular, “right” way to conduct qualitative projects. Such concerns are often amplified by fear that deviation from rigid adherence to established sets of procedures may jeopardize the (perceived or actual) rigor of the work. While the appeal of recipe-like means of ensuring rigor is understandable, fixation on compliance with “established” approaches overlooks the fact that versions of recognizable, named approaches (e.g., grounded theory) often use *different* procedures [7]. As Braun and Clarke suggest, this “hallowed quest” for a singular, ideal approach leads many researchers astray and risks limiting appropriate and necessary adaptations and innovations in methods [13]. IS researchers seeking to broaden the range of approaches they can apply should take comfort that there is “no single right way to do qualitative data analysis […]. Much depends on the purpose of the research, and it is important that the proposed method of analysis is carefully considered in planning the research, and is integrated from the start with other parts of the research, rather than being an afterthought.” [14]. At the same time, given the wealth of traditions represented in the IS community, it can be difficult for researchers to effectively ensure and convey the quality and rigor of their work. This paper aims to serve as a resource for IS researchers seeking innovative and accessible approaches to qualitative research. We present suggestions for developing and communicating approaches to analysis that are the right “fit” for complex IS research projects and demonstrate rigor and quality.

Accordingly, section 1 offers guidance on identifying an analytic approach that aligns with study goals and allows for practical constraints. We describe three approaches commonly considered for IS projects: grounded theory, framework analysis, and interpretive phenomenological analysis, highlighting core elements that researchers can borrow to create a tailored, pragmatic approach. Section 2 addresses opportunities to ensure and communicate the rigor of pragmatic analytic approaches. Section 3 provides an illustrative example from the team’s work, describing the design and execution of a pragmatic analytic approach and the diversity of research and practice products generated.

## Section 1: ensuring fit between research goals, practical constraints, and analytic approaches

**Fig. 1:**
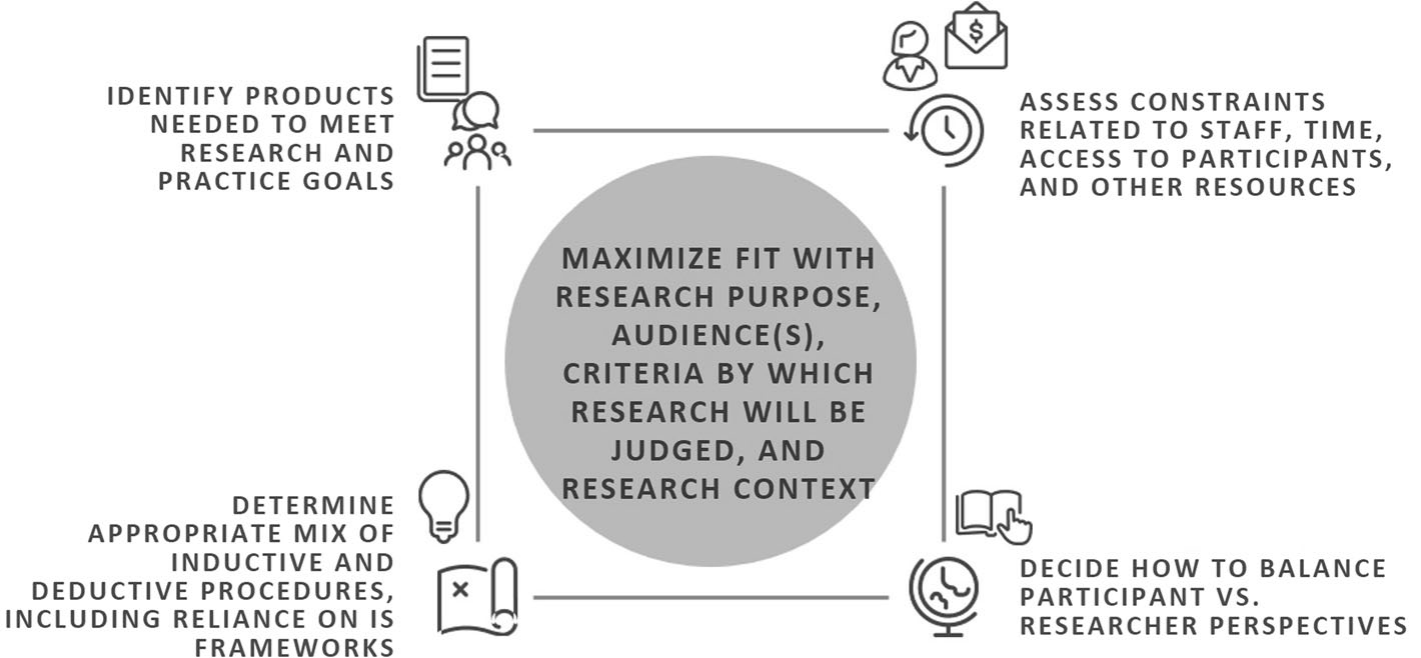
Developing a pragmatic qualitative data analysis approach for IS: key considerations for selection of analytic procedures

Decision-making about all aspects of research design, including analysis, entails judgment about “fit.” Researchers need not identify a single analytic approach and attempt to force its strict application, regardless of fit. Indeed, the flexible, study-specific combination of design elements is a hallmark of applied qualitative research practice [9]. Relevant considerations for fit include the inquiry’s purpose and nature of the subject matter; the diversity of intended audiences for findings; the criteria used to judge the quality and practical value of the results; and the research context (including characteristics of the setting, participants, and investigators). Other important considerations relate to constraints of available resources (e.g., funding, time, and staff) and access to relevant participants [3]. We contend that in the applied IS setting, finding an appropriate fit often includes borrowing procedures from different approaches to create a pragmatic, hybrid approach. A pragmatic approach also addresses the IS-specific tensions outlined above, i.e., a need to conduct research that is time-bounded, engages with theories/frameworks/models, supports application in practice, and speaks to a diversity of colleagues. To promote goals of achieving fit and internal coherence in light of IS-specific requirements, we offer the considerations above and additional guiding questions for selecting analytic procedures to create a pragmatic approach, as summarized in Fig. 1.

Key questions include the following:

1. What is the appropriate balance of inductive and deductive analytic procedures given the research goals?

A deductive process emphasizes themes and explanations derived from previously established concepts, pre-existing theories, or the relevant literature [9]. For example, an analysis that leans heavily on a deductive process might use the core components of the Exploration, Preparation, Implementation, Sustainment (EPIS) framework [15] to inform the coding structure and analysis. This process would support efforts to bound the investigation’s scope or expand an existing framework or model [16]. On the other hand, rather than trying to fit data with pre-existing concepts or theory, an inductive process generates interpretation and understanding that is primarily grounded in and driven by the data [9].

A balance of deductive and inductive processes might use an IS framework as a starting point for the deductive portion and then emphasize inductive processes to garner additional insight into topics not anticipated by the team or framework. For example, a selected IS framework may not attend sufficiently to the ways in which implementation context drives inequities [17], if the dataset includes valuable information on this topic, including inductive processes would allow a fuller exploration of such patterns.

2. To what extent will the analysis emphasize the perspectives of participants vs. researchers?

An important decision relates to where the research team wishes to ground the analysis on the continuum between insider (emic) and outsider (etic) perspectives. The appropriate balance of insider/outsider orientation will reflect the overall research design and questions. Specific decisions about how to execute the desired balance through the *analysis* include; for example, the types of codes used or the value placed on participant reflections. As described below in section 2, value is often placed on incorporating participants’ feedback on the development analysis, sometimes called “member checks” or “member reflections” [8].

An insider (emic) orientation represents findings in the ways that participants experience them, and insider knowledge is valued and privileged [9]. As an example, MacFarlane and colleagues used Normalization Process Theory and participatory approaches to identify appropriate implementation strategies to support the integration of evidence-based cross-cultural communication in European primary care settings. The participatory nature of the project offered the opportunity to gain “insider” insight rather than imposing and prioritizing the academic researchers’ “outsider” perspective. The insider (emic) orientation was operationalized in the analytic approach by using stakeholder co-analysis, which engages a wider set of stakeholders in the iterative processes of thematically analyzing the data [18]. By contrast, an outsider (etic) orientation represents the setting and participants in terms that the researcher or external audiences bring to the study and emphasizes the outsider’s perspective [9]. For instance, Van deGriend and colleagues conducted an analysis of influences on scaling-up group prenatal care. They used outsider (etic) codes that drew on researchers’ concepts and the literature to complement the insider (emic) codes that reflected participants’ concepts and views [19]. Balancing insider and outsider orientations is useful for pragmatic, qualitative IS studies increase the potential for the study to highlight practice- and community-based expertise, build the literature, and ultimately support the integration of evidence into practice.

3. How can the analytic plan be designed to yield the outputs and products needed to support the integration of evidence into research and practice?

The research team can maximize efficiency and impact by intentionally connecting the analytic plan and the kind of products needed to meet scientific and practice goals (e.g., journal articles versus policy briefs). The ultimate use of the research outputs can also impact decisions around the breadth versus depth of the analysis. For example, in a recent implementation evaluation for community-clinical partnerships delivering EBIs in underserved communities, members of this author team (SR and RL) analyzed data to explore how partnership networks impacted implementation outcomes. At the same time, given the broader goal of supporting the establishment of health policies to support partnered EBI delivery, the team was also charged (by the state Department of Public Health) with capturing stories that would resonate with legislators regarding the need for broad, sustained investments [20]. We created a unique code to identify these stories during analysis and easily incorporate them into products for health department leaders. Given the practice-focused orientation, qualitative IS studies often support products for practitioners, e.g., “playbooks” to guide the process of implementing an intervention or novel care process [1].

4. How can analysis resources be used strategically in time-sensitive projects or where there is limited staff or resource availability?

IS research is often conducted by teams, and strategic analytic decisions can promote rigor while capitalizing on the potential for teamwork to speed up analysis. Deterding and Waters’ strategy of flexible coding, for example, offers such benefits [21]. Through an initial, framework-driven analytic step, large chunks of text can be quickly indexed deductively into predefined categories, such as the five Consolidated Framework for Implementation Research domains of inner setting, outer setting, characteristics of individuals, intervention attributes, and processes [22]. This is a more straightforward coding task appropriate for research assistants who have been trained in qualitative research and understand the IS framework. Then, during the second analytic coding step, more in-depth coding by research team members with more experience can ensure a deeper exploration of existing and new themes. This two-step process can also enable team members to lead different parts of an IS project with different goals, purposes, or audiences. Other innovations in team-based analyses are becoming increasingly common in IS, such as rapid ethnographic approaches [23].

### Building blocks for pragmatic analysis: examples from pattern-based analytic approaches

We offer illustrative examples of established analytic approaches in the following, highlighting their utility for IS and procedures that a pragmatic approach might usefully borrow and combine. These examples are not exhaustive; instead, they represent selected, pattern-based analytic approaches commonly used in IS. We aim to offer helpful anchor points that encompass the breadth and flexibility to apply to a wide range of IS projects [24] while also reflecting and speaking to a diversity of home disciplines, including sociology, applied policy, and psychology.

#### Grounded theory

Grounded theory is one of the most recognizable and influential approaches to qualitative analysis, although many variations have emerged since its introduction. Sociologists developed the approach, and the history and underlying philosophy are richly detailed elsewhere [25, 26]. The central goal of this approach is to generate a theoretical explanation grounded in close inspection of the data and without a preconceived starting point. In many instances, the emphasis of grounded theory on a purely inductive orientation may be at odds with the focus in IS on the use of existing theories and frameworks, as highlighted by the QUALRIS group [4]. Additionally, a “full” grounded theory study, aligned with all its methodological assumptions and prescriptions (e.g., for sampling), is very demanding and time-consuming and may not be appropriate when timely turnaround in the service of research or practice change is required. For these reasons, a full grounded theory approach is rarely seen in the IS literature. Instead, IS researchers who use this approach are likely to use a modified version, sometimes described as “grounded theory lite” [6].

Core features and procedures characteristic of grounded theory that can be incorporated into a pragmatic approach include inductive coding techniques [27]. Open, inductive coding allows the researcher to “open up the inquiry” by examining the data to see what concepts best fit the data, without a preconceived explanation or framework [28–30]. Concepts and categories derived from open coding prompt the researcher to consider aspects of the research topic that were overlooked or unanticipated [31]. The intermediate stages of coding in grounded theory, referred to as axial or focused coding, build on the open coding and generate a more refined set of key categories and identify relationships between these categories [32]. Another useful procedure from grounded theory is the constant comparison method, in which data are collected, categorized, and compared to previously collected data. This continuing, iterative process prompts continuous engagement with the analysis process and reshapes and redefines ideas, which is useful for most qualitative studies [25, 29, 33]. Grounded theory also allows for community expertise and broader outsider perspectives to complement one another for a more comprehensive understanding of practices [34].

An illustration of the utility of grounded theory procedures comes from a study that explored how implementing organizations can influence local context to support the scale-up of mental health interventions in middle-income countries [35]. Using a multiple case study design, the study team used an analytic approach based on grounded theory to analyze data from 159 semi-structured interviews across five case sites. They utilized line-by-line open coding, constant comparison, and exploration of connections between themes in the process of developing an overarching theoretical framework. To increase rigor, they employed triangulation by data source and type and member reflections. Their team-based plan included multiple coders who negotiated conflicts and refined the thematic framework jointly. The output of the analysis was a model of processes by which entrepreneurial organizations could marshal and create resources to support the delivery of mental health interventions in limited-resource settings. By taking a divergent perspective (grounded in social entrepreneurship, in this case), the study output provided a basis for further inquiry into the design and scale-up of mental health interventions in middle-income countries.

#### Framework analysis

Framework analysis comes from the policy sphere and tends to have a practical orientation; this applied nature typically includes a more structured and deductive approach. The history, philosophical assumptions, and core processes are richly described by Ritchie and Spencer [36]. Framework analysis entails several features common to many qualitative analytic approaches, including defining concepts, creating typologies, and identifying patterns and relationships, but does so in a more predefined and structured way [37, 38]. For example, the research team can create codes based on a framework selected in advance and can also include open-ended inquiry to capture additional insights. This analytic approach is well-suited to multi-disciplinary teams whose members have varying levels of experience with qualitative research [37]. It may require fewer staff resources and less time than some other approaches.

The framework analysis process includes five key steps. Step 1 is familiarization: Team members immerse themselves in the data, e.g., reading, taking notes, and listening to audio. Step 2 is identifying a coding framework: The research team develops a coding scheme, typically using an iterative process primarily driven by deductive coding (e.g., based on the IS framework). Step 3 is indexing: The team applies the coding structure to the entire data set. Step 4 is charting: The team rearranges the coded data and compares patterns between and within cases. Step 5 is mapping and interpretation: The team looks at the range and nature of relationships across and between codes [36, 39, 40]. The team can use tables and diagrams to systematically synthesize and display the data based on predetermined concepts, frameworks, or areas of interest. While more structured than other approaches, framework analysis still presents a flexible design that combines well with other analytic approaches to achieve study objectives [37]. The case example given in section 3 offers a detailed application of a modified framework analytic approach.

#### Interpretive phenomenological analysis (IPA)

Broadly, the purpose of a phenomenological inquiry is to understand the experiences and perceptions of individuals related to an occurrence of interest [41, 42]. For example, a phenomenological inquiry might focus on implementers’ experiences with remote training to support implementing a new EBI, aiming to explore their views, how those changed over time, and why implementers reacted the way they did. Drawing on this tradition, IPA focuses specifically on particular individuals (or cases), understanding both the experience of individuals and the sense they are making of those experiences. With roots in psychology, this approach prioritizes the perspective of the participant, who is understood to be part of a broader system of interest; additional details about the philosophical underpinnings are available elsewhere [41]. Research questions are open and broad, taking an inductive, exploratory perspective. Samples are typically small and somewhat homogeneous as the emphasis is placed on an in-depth exploration of a small set of cases to identify patterns of interest [43]. Despite the smaller sample size, the deep, detailed analysis requires thoughtful and time-intensive engagement with the data. The resulting outputs can be useful to develop theories that attend to a particular EBI or IS-related process or to refine existing frameworks and models [44].

A useful example comes from a study that sought to understand resistance to using evidence-based guidelines from the perspective of physicians focused on providing clinical care [45]. The analysis drew on data collected from interviews of 11 physicians selected for their expertise and diversity across a set of sociodemographic characteristics. In the first phase of the analysis, the team analyzed the full-length interviews and identified key themes and the relationships between them. Particular attention was paid to implicit and explicit meanings, repeated ideas or phrases, and metaphor choices. Two authors conducted the analyses separately and then compared them to reach a consensus. In the second phase of the analysis, the team considered the group of 11 interviews as a set. Using an inductive perspective, the team identified superordinate (or high-level) themes that addressed the full dataset. The final phase of the analysis was to identify a single superordinate theme that would serve as the core description of clinical practice. The team engaged other colleagues from diverse backgrounds to support reflection and refinement of the analysis. The analysis yielded a theoretical model that focused on a core concept (clinical practice as engagement), broken out into five constituent parts addressing how clinicians experience their practice, separate from following external guidelines.

## Section 2: ensuring and communicating rigor of a pragmatic analysis

Building on the discussion of pragmatic combination of approaches for a given study, we turn now to the question of ensuring and communicating rigor so that consumers of the scientific products will feel confident assessing, interpreting, and engaging with the findings [46]. This is of particular importance for IS given that the field tends to emphasize quantitative methods and there may be perceptions that qualitative research (and particularly research that must be completed more quickly) is less rigorous. To address those field-specific concerns and ensure pragmatic approaches are understood and valued, IS researchers must ensure and communicate the rigor of their approach. Given journal constraints, authors may consider using supplementary files to offer rich details to describe the study context and details of coding and analysis procedures (see for example, Aveling et al. [47]). We build on the work of Mays and Pope [38], Tracy [8], and others [48–52] to offer a shortlist of considerations for IS researchers to ensure pragmatic analysis is conducted with rigor and its quality and credibility are communicated (Table 1). We also recommend these articles as valuable resources for further reading.

**Table 1:** Suggestions to ensure and communicate rigor in pragmatic qualitative analysis for IS

Consideration	Description
Demonstrate the link between research goals, analytic approach, findings, and broader literature	Researchers should explain how and why they are incorporating procedures from different approaches. By explicitly justifying their decisions and connecting these pieces of the overall research design, the team can ensure internal coherence as they combine procedures from approaches that may have distinct underlying principles and assumptions.
Ensure transparency around data analysis	Researchers should provide sufficient details about which procedures from which analytic approaches have been used and how they were combined or adapted to enable readers and users of the research to understand and evaluate the utility of the work. Details may include, e.g., the initial coding structures and how conceptual frameworks influenced analysis. Additionally, for data collected among diverse participant groups (e.g., EBI recipients vs. implementers) or sites, details about if/how data were analyzed separately and then holistically are critical. Ongoing documentation of the analytic process, including description of decision-making and mediation of disagreements, also supports transparent reporting.
Triangulate data	The analysis can be strengthened by comparing results from different methods of inquiry (e.g., participant observation and focus group discussions) or different sources (e.g., implementers and leaders) to gain a more comprehensive and nuanced view of the IS concerns at hand.
Integrate reflexivity	The researchers should describe how their background, experience, and positions (particularly in terms of being grounded in research or practice) may influence their analysis of the data. Relevant details may include experience with the implementation effort, setting, implementers, and EBI of interest.
Use member reflections	Sharing early findings with members of participant groups to get feedback offers an opportunity to strengthen the analysis and help meet practice goals. This could include sharing early interpretations with an advisory group or key implementation stakeholders to gather suggestions to further refine/develop analyses.
Consider divergent cases	It is important to identify and investigate not only the broadly consistent themes but the deviant cases as well. This ensures a wide range of explanations have been considered, and the bulk of the cases have been included in the summaries offered. For example, this might prompt attention to an implementation site with a vastly different experience implementing a new innovation compared to others in its network.

Reporting checklists can help researchers ensure the details of the pragmatic analytic approach are communicated effectively, and inclusion of such a checklist is often required by journals for manuscript submission. Popular choices include the Standards for Reporting Qualitative Research (SRQR) and Consolidated Criteria for Reporting Qualitative (COREQ) checklists. These were developed based on reviews of other checklists and are intended to capture a breadth of information to increase transparency, rather than being driven by a philosophical underpinning regarding how to design rigorous qualitative research [53, 54]. For that reason, researchers should use these checklists with a critical lens as they do not alone demonstrate rigor. Instead, they can be thought of as a flexible guide and support, without focusing solely on technical components at the expense of the broader qualitative expertise that drives the research effort [55].

## Section 3: case example of a modified framework analysis approach

To illustrate the ideas presented above, we offer a recent example of work conducted by two authors (AR and SR) and colleagues [56]. The broad motivation for the study was to increase the use of EBIs in community-based organizations (CBOs) and faith-based organizations (FBOs) working with underserved communities. Our past work and the literature highlighted challenges in matching practitioner capacity (i.e., knowledge, motivation, skills, and resources) with the skillset required to use EBIs successfully [57, 58]. The study utilized a participatory implementation science perspective, which offered a unique opportunity to integrate insider and outsider perspectives and increase the likelihood that solutions developed would reflect the realities of practice. The work was conducted in partnership with a Community Advisory Board and attempted to balance research and action [59, 60].

The qualitative portion of the project had two primary goals. The research goal was to identify improvements to the design and delivery of capacity-building interventions for CBOs and FBOs working with underserved populations. The practice-related goal was to identify local training needs and refine an existing EBI capacity-building curriculum. We drew on the EPIS Framework [15] to support our exploration of multi-level factors that drive EBI implementation in social service settings. We conducted four focus group discussions with intended capacity-building recipients (*n* = 27) and key informant interviews with community leaders (*n* = 15). Given (1) the applied nature of the research and practice goals, (2) our reliance on an existing IS framework, (3) limited staff resources, and (4) a need to analyze data rapidly to support intervention refinement, we chose a modified framework analysis approach. Modifications included incorporating aspects of grounded theory, including open coding, to increase the emphasis on inductive perspectives. The team also modified the charting procedures, replacing tabular summaries with narrative summaries of coded data.

Analysis was conducted by three doctoral-level researchers with complementary training (IS, sociology, and nursing). We started by familiarizing ourselves with the data — the three researchers read a subset of the transcripts, with purposeful overlap in reading assignments to facilitate discussion. Then, we created the coding framework and indexed the data. We went back and forth between indexing and charting, starting with deductive codes based on the EPIS framework, and then using a more inductive open coding strategy to identify emergent codes that fell outside the EPIS framework, e.g., the importance of investing in resources that remain in the community. The new coding framework, with both inductive and deductive codes, was applied to all interview transcripts. Each transcript was independently coded by two of the three investigators, followed by coding comparison to address discrepancies. We used NVivo 12 software [61], which enabled the exploration and reorganization of data to examine patterns within specific codes and across the data set. We utilized narrative summaries to organize our findings. Finally, we revisited the relevant data to identify broad themes of interest. This step was collaborative and iterative, with each team member taking the lead on a subset of codes and themes that aligned with their expertise, and the interpretations were shared with the other research investigators and discussed. This “divide-and-conquer” tactic was similar to the Deterding and Waters example of flexible coding [21]. We used triangulation to explore perceptions by different groups of participants (e.g., leaders vs. program implementers and individuals representing CBOs vs. FBOs). This type of triangulation is sometimes referred to as “triangulation of data” and stands in contrast to triangulation between different methods [62].

Our analytic plan was informed by the participatory design of the larger project. At multiple points in the analytic process, we presented interpretations to the advisory board and then refined interpretations and subsequent steps of the analysis accordingly. This was critical because our use of an IS framework likely imposed an outsider’s perspective on the use of EBIs in practice and we wanted to ensure the interpretations reflected insider perspectives on the realities of practice. The incorporation of practice-based expertise in our analytic process also reflected the participatory nature of the research project. We note that advisory board members did not wish to analyze the data in-depth and instead preferred this manner of engagement.

To meet our research goals, we produced scientific publications that expanded the literature on capacity-building strategies to promote evidence-based prevention in CBOs and FBOs addressing health equity. The modified framework analysis approach allowed us to build on and extend the EPIS framework by allowing for framework-driven deductive coding and open, inductive coding. As an example, the EPIS framework highlights relationships between patient/client characteristics (within the “outer context” domain) and EBI fit (within the “innovation” domain). We added an emergent code to capture the wide range of resources CBO- and FBO-based practitioners needed to improve the fit between available EBIs and community needs. This included attention to the limitations of available EBIs to address the multi-level barriers to good health experienced by underserved communities. Participants highlighted the importance of solutions to these gaps coming not from external resources (such as those highlighted within the “bridging factors” domain of the framework), but instead from resources built and maintained within the community. Per the journal’s requirements, we presented the SRQR checklist to explain how we ensured a rigorous analysis.

To achieve practice goals, we drew on the rich dataset to refine the capacity-building intervention, from recruitment to the training components and ongoing supports. For example, we were able to create more compelling arguments for organizational leaders to send staff to the training and support the use of EBIs in their organizations, use language during trainings that better resonated with trainees, and include local examples related to barriers and facilitators to EBI use. We also revised programmatic offerings to include co-teaching by community members and created shorter, implementation-focused training opportunities. The balance of framework-driven, deductive processes, and open, inductive processes allowed us to capture patterns in anticipated and unanticipated content areas. This balance also allowed us to develop research briefs that provide high-level summaries that could be useful to other practitioners considering how best to invest limited professional development resources.

## Conclusions

We encourage IS researchers to explore the diversity and flexibility of qualitative analytic approaches and combine them pragmatically to best meet their needs. We recognize that some approaches to analysis are tied to particular methodological orientations and others are not, but a pragmatic approach can offer the opportunity to combine analytic strategies and procedures. To do this successfully, it is essential for the research team to ensure fit, preserve quality, and rigor, and provide transparent explanations connecting the analytic approach and findings so that others can assess and build on the research. We believe pragmatic approaches offer an important opportunity to make strategic analytic decisions, such as identifying an appropriate balance of insider and outsider perspectives, to extend current IS frameworks and models. Given the urgency to increase the utilization and utility of EBIs in practice settings, we see a natural fit with the pragmatist prompt to judge our research efforts based on whether or not the knowledge obtained serves our purposes [63]. In that spirit, the use of pragmatic approaches can support high-quality, efficient, practice-focused research, which can broaden the scope and ultimate impact of IS research.

## Data Availability

Not applicable
